# Sulforaphane attenuates aldose reductase-mediated platelet dysfunction in high glucose-stimulated human platelets via downregulation of the Src/ROS/p53 signaling pathway

**DOI:** 10.3389/fnut.2025.1663245

**Published:** 2025-08-01

**Authors:** Xiaoyan Bi, Xinhui Huang, Chunmei Zhang, Xin Zhao, Junyu Ma, Mengyao Li, Xuexun Li, Bangzhao Zeng, Rong Li, Xian Zhang, Fuli Ya

**Affiliations:** ^1^Department of Nutrition, School of Public Health, Dali University, Dali, Yunnan, China; ^2^Huzhou Health Vocational College, Huzhou, Zhejiang, China; ^3^Department of Laboratory Teaching Center, School of Public Health, Dali University, Dali, Yunnan, China; ^4^Department of Cardiology, Dali Bai Autonomous Prefecture People's Hospital, Dali, Yunnan, China

**Keywords:** sulforaphane, platelet hyperreactivity, apoptosis, high glucose, aldose reductase, p53

## Abstract

**Background:**

Platelet abnormalities are well-recognized complications of type 2 diabetes mellitus (T2DM). High glucose (HG) increases platelet mitochondrial dysfunction, apoptosis and hyperreactivity in T2DM, which underlie the occurrence of thrombotic events. Sulforaphane (SFN) is a dietary isothiocyanate enriched in cruciferous vegetables and possesses multiple biological activities. This study aimed to explore the efficacy of SFN on platelet dysfunction in HG-stimulated human platelets *in vitro*.

**Methods:**

Washed human platelets from healthy donors were pre-incubated with SFN (5, 10, or 20 μM) or vehicle control (0.05% DMSO) for 40 min at 37°C, with or without pharmacologic inhibitors (apalrestat, PP2, N-acetyl-cysteine, pifithrin-μ). Platelets were then stimulated with normal glucose (NG, 5 mM) or HG (25 mM) for an additional 90 min. Functional assays were performed to evaluate SFN efficacy and investigate its underlying mechanisms.

**Results:**

The results demonstrated that SFN attenuated HG-induced platelet dysfunction by alleviating mitochondrial dysfunction (manifested as loss of mitochondrial membrane potential; *p* < 0.001), apoptosis (characterized by increased caspase-9/-3 activation and phosphatidylserine exposure; *p* < 0.01), and hyperreactivity (evidenced by enhanced aggregation and activation; *p* < 0.05). Mechanistically, SFN significantly suppressed HG-induced aldose reductase (AR) activity (*p* < 0.001). Pharmacological inhibition revealed that the beneficial effects of SFN on platelet function were mediated mechanistically through AR downregulation, which attenuated p53 phosphorylation via Src-dependent ROS generation.

**Conclusion:**

These findings suggest that by inhibiting the Src/ROS/p53 signaling pathway and mitigating AR-mediated platelet dysfunction, SFN may confer significant protection against atherothrombosis during hyperglycemia.

## 1 Introduction

Diabetes mellitus (DM) has become a significant global public health challenge (1). In China, the number of individuals diagnosed with DM increased markedly from 90 million to 140 million between 2011 and 2021, posing a formidable challenge for disease prevention and control (2). Type 2 diabetes mellitus (T2DM) represents the predominant form, accounting for the majority of cases. Approximately 75–80% of T2DM patients develop cardiovascular complications (3). Among these complications, atherosclerosis and thrombotic events (such as myocardial infarction and stroke) are responsible for approximately 80% of cardiovascular-related deaths (4), highlighting the severe threat T2DM poses to cardiovascular health. Platelets not only play crucial roles in physiological hemostasis, but also contribute to pathological thrombosis and coagulopathy (5). Under diabetic conditions, platelets exhibit hyperactivation, which serves as a key contributor to accelerated vascular inflammation and thrombosis (6). In diabetes, dysregulation of glycolipid metabolism, oxidative stress and chronic inflammation can promote platelet hyperreactivity and foster pathological thrombus formation (7, 8). Therefore, controlling excessive platelet activation represents a critical therapeutic strategy for preventing and managing diabetes-associated complications (9).

Mechanisms that contribute to platelet dysfunction in T2DM are complicated and poorly understood. It has been evidenced that aldose reductase (AR) plays a central role in platelet abnormalities and thrombus formation during the progression of T2DM (8, 10). Under normal physiological conditions, the polyol pathway mediated by AR accounts for a relatively low proportion of glucose metabolism. Nevertheless, under hyperglycemic conditions, enhanced AR activity can augment the contribution of the polyol pathway to glucose metabolism by approximately 25–30%. The abnormal activation of this pathway exacerbates oxidative stress and contributes to a variety of diabetes-associated complications (11). It has been reported that high level of glucose (HG) and collagen exposure promote platelet hyperreactivity via AR-mediated thromboxane generation (10). AR-mediated ROS generation and p53 phosphorylation leads to mitochondrial dysfunction and damage in diabetic platelets, which contributes to platelet hyperreactivity, apoptosis, and thrombus formation (8). Thus, targeting AR and its downstream signaling pathways have been considered to be promising approaches to normalize platelet function and allow for effective thrombolysis and thromboprophylaxis under hyperglycemic conditions (12).

Diet or dietary composition intervention holds significant importance in the prevention and treatment of cardiovascular diseases (CVDs) (13–15). Sulforaphane (SFN) is a dietary isothiocyanate abundant in cruciferous vegetables such as broccoli, cauliflower, kohlrabi, and mustard (16, 17). Extensive *in vitro* studies, animal models, and clinical trials have demonstrated that SFN possesses anti-inflammatory, antioxidant, anticancer, and cardioprotective properties (18–20). We and others previously demonstrated that SFN attenuated platelet mitochondrial dysfunction and platelet activation induced by physiological agonists and oxidized low-density lipoprotein (ox-LDL) (20–23). Moreover, SFN has been demonstrated to improve glucose metabolism, reduce insulin resistance, and possess protective effects against diabetes complications (24). However, the role of SFN in platelet dysfunction under hyperglycemic conditions has never been explored. Therefore, the present study aimed to investigate the protective effect of SFN against HG-induced platelet dysfunction and to elucidate the underlying mechanisms in human platelets *in vitro*.

## 2 Materials and methods

### 2.1 Chemicals and reagents

L-sulforaphane (purity ≥ 95%), glucose and *N*-acetyl-cysteine (NAC) were purchased from Sigma-Aldrich (MO, United States). Collagen was obtained from Chrono-Log Corp. (PA, United States). Apalrestat (ARI) and pifithrin-*μ* (PFT-μ) were purchased from Selleck Chemicals (TX, United States). Antibodies against Bax, Bcl-xL, cleaved-caspase-3 (17 kDa), cleaved-caspase-9 (37 kDa), phospho-p38α MAPK, β-actin, GAPDH and secondary antibodies (goat anti-rabbit or anti-mouse IgG-HRP) were purchased from Bioworld Technology, Inc. (MN, United States). Antibody against phospho-p53 was purchased from OriGene (MD, USA).

### 2.2 Human subject recruitment

Thirty healthy adults (aged 20–40 years) without history of diseases known to affect platelet function (e.g., hyperlipidemia, DM, CVDs, hemostatic disorders), free of anti-platelet medications for ≥14 days, and without being taking dietary supplements were recruited for this investigation (20, 23). The Ethics Committee of Dali University approved the study protocol. Prior to enrollment, all participants provided written informed consent in accordance with institutional guidelines and the Declaration of Helsinki.

### 2.3 Human blood collection and platelet preparation

Human blood collection and washed platelet preparation were conducted in accordance with our previously described methods (20, 23). In brief, 20 mL of whole blood anticoagulated with 3.8% sodium citrate (1/9, vol/vol) was collected from the cubital veins of volunteers following an overnight fast, with sampling conducted during morning hours (08.00–10:00). Platelet-rich plasma (PRP) was isolated by centrifuging citrated blood at 150 × *g* for 20 min at 37°C and subsequently diluted threefold in acid citrate dextrose (ACD; 147 mM glucose, 39 mM citric acid, 75 mM trisodium citrate, pH 6.5) containing 0.1 U/mL apyrase. Washed platelets were prepared by centrifuging PRP at 800 × *g* for 10 min. The resulting platelet pellet was resuspended in Tyrode’s buffer (137 mM NaCl, 2 mM KCl, 1 mM MgCl₂, 0.34 mM NaH₂PO₄, 12 mM NaHCO₃, 5.5 mM glucose, 5 mM HEPES, pH 7.4) and adjusted to a final concentration of 3.0 × 10^8^ platelets/mL.

### 2.4 Platelet aggregation assay

The platelet aggregation rate was measured by the turbidimetric method, as we previously described (20, 23). Human washed platelets (3.0 × 10^8^/mL) were pre-incubated with different concentrations of SFN (5, 10, or 20 μM) or vehicle control (0.05% DMSO) for 40 min at 37°C with or without pharmacologic inhibitors such as ARI (10 μM), PP2 (20 μM), NAC (500 μM), and PFT-μ (20 μM), followed by the stimulation of normal glucose (NG; 5 mM) or HG (25 mM) for additional 90 min. In the presence of 1 mM CaCl₂, platelet aggregation stimulated by 1 μg/mL collagen was detected by using a Techlink 400 aggregometer (Techlink Biomedical Technology Corp., Beijing, China). The 25 mM glucose concentration is commonly employed to simulate hyperglycemia in diabetic platelet models, as evidenced by prior mechanistic studies assessing platelet hyperreactivity under sustained high-glucose conditions (7, 8, 10).

### 2.5 Measurement of platelet surface CD62P expression

Platelet surface expression of CD62P, an established marker of platelet activation, was quantified by flow cytometry (FACSCalibur; BD Biosciences, CA, United States) in human washed platelets (5 × 10^6^/mL) (20, 23). Briefly, platelets were pretreated with varying SFN concentrations (5, 10, or 20 μM) or vehicle control for 40 min at 37°C in the presence or absence of specific inhibitors. Subsequently, cells were stimulated for an additional 90 min with either NG (5 mM) or HG (25 mM). Following stimulation, platelets were incubated with a PE-conjugated anti-human CD62P antibody for 20 min at room temperature in the dark. Samples were then fixed using 1% paraformaldehyde prior to flow cytometric analysis. The data were analyzed by using FlowJo software (Version 10.8.1, Tree Star Inc., OR, United States).

### 2.6 Measurement of mitochondrial membrane potential (ΔΨm) and phosphatidylserine (PS) exposure

Human washed platelets (5 × 10^6^/mL) were pre-incubated with different concentrations of SFN or vehicle control for 40 min at 37°C with or without pharmacologic inhibitors, followed by the stimulation of NG (5 mM) or HG (25 mM) for additional 90 min. Platelet ΔΨm was then assessed using the red-fluorescent probe tetramethylrhodamine methyl ester (TMRM; Abcam, Cambridge, United Kingdom), following our previously described methods (23). TMRM accumulation within functional mitochondria yields a fluorescent signal that diminishes upon dissipation of ΔΨm. Moreover, an Annexin V PE/7-AAD apoptosis assay kit (Solarbio Life Sciences, Beijing, China) was used to measure the levels of platelet PS exposure (23). Platelet ΔΨm and PS exposure were measured by using a FACSCalibur flow cytometer.

### 2.7 Measurement of intraplatelet ROS levels

The DCFDA/H2DCFDA cellular ROS assay kit (Abcam, Cambridge, United Kingdom) was used to detect the total ROS levels in platelets. Briefly, SFN-pretreated human washed platelets (5 × 10^5^/mL) were stimulated with NG (5 mM) or HG (25 mM) for additional 90 min, followed by the addition of 20 μM DCFDA for 30 min at 37°C in the dark. The total ROS levels in platelets were measured using a flow cytometer.

### 2.8 Western blotting analysis

Western blot analysis was carried out as we previously described (20, 23, 25). Briefly, protein concentrations in total cell lysates were determined using a BCA assay kit (Beyoutime, Shanghai, China). Subsequently, 20 μg of protein per sample were resolved by sodium dodecyl sulfate-polyacrylamide gel electrophoresis (SDS-PAGE) and electrotransferred onto polyvinylidene difluoride (PVDF) membranes. Membranes were blocked with 5% bovine serum albumin (BSA) for 1 h and then probed overnight at 4°C with primary antibodies. Membranes were then incubated with appropriate horseradish peroxidase (HRP)-conjugated secondary antibodies. Protein bands were visualized using an enhanced chemiluminescence (ECL) detection system. Band intensity quantification was performed via grayscale analysis using ImageJ software (version 1.37v; NIH, United States).

### 2.9 Measurement of AR activity

The AR activity was determined using a commercial Aldose Reductase Activity Kit (Abcam, Cambridge, United Kingdom), according to the manufacturer’s protocols. Briefly, the platelets were lysed and centrifuged as for Western blot analysis. The supernatant was used for the measurements of AR activity. AR activity was assayed in a 1 mL reaction mixture (0.4 M Li₂SO₄, 0.1 mM NADPH, 10 mM DL-glyceraldehyde, and 0.05 mM potassium phosphate, pH 6.0). Activity was monitored at room temperature by measuring the absorbance change at 340 nm (10).

### 2.10 Statistical analysis

Data were presented as mean ± standard error of the mean (SEM) from a minimum of three independent biological replicates. Statistical analyses utilized GraphPad Prism (version 9.4.1; GraphPad Software, San Diego, CA). Intergroup differences were assessed using a one-way analysis of variance (ANOVA) with Dunnett’s *t*-test or Tukey’s *post hoc* test for multiple comparisons. Statistical significance was defined as *p* < 0.05.

## 3 Results

### 3.1 SFN attenuates HG-induced platelet mitochondrial dysfunction, apoptosis, and hyperreactivity

**Figure 1:**
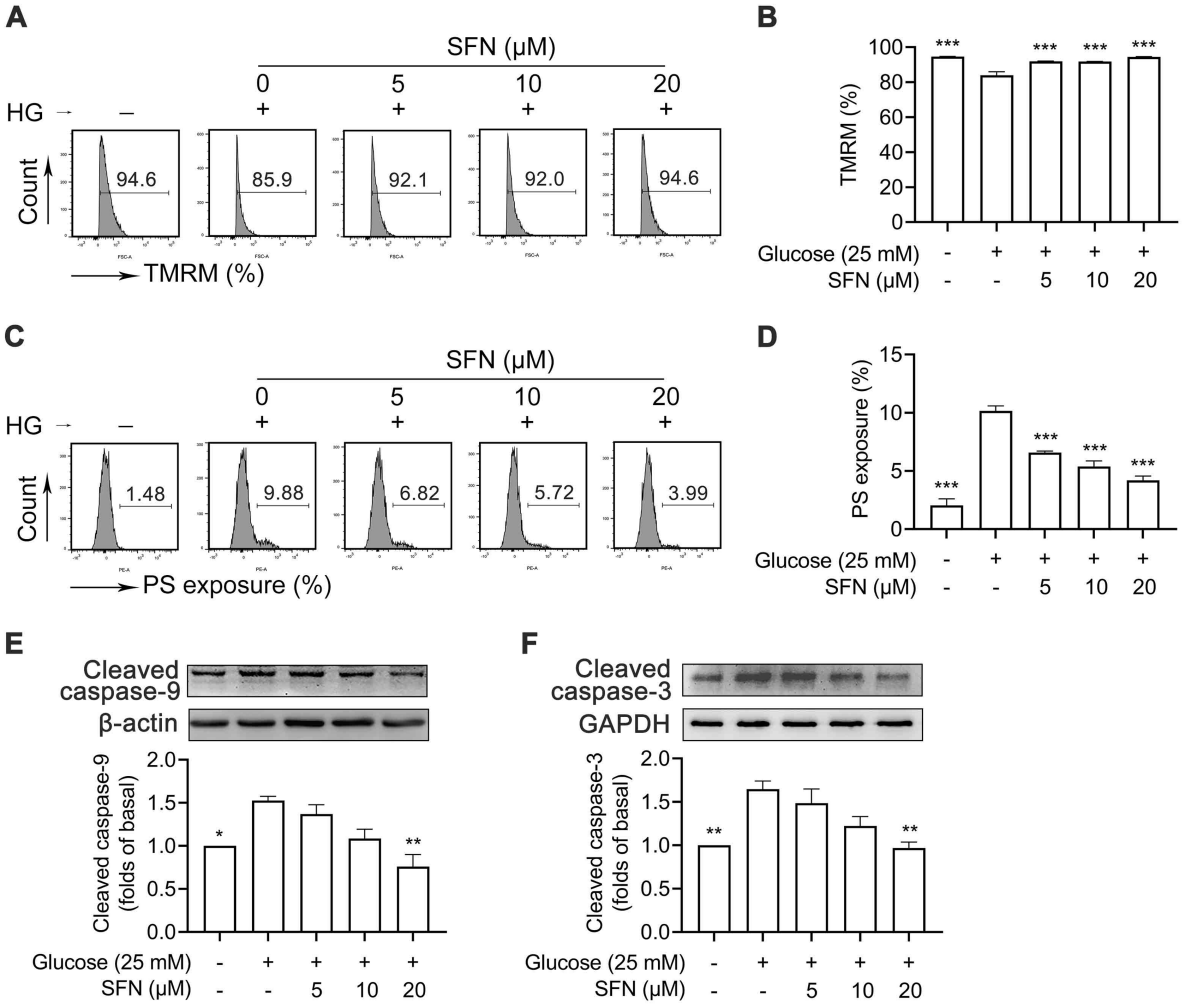
Sulforaphane (SFN) attenuates HG-induced platelet mitochondrial dysfunction and apoptosis. Washed human platelets were pre-incubated with SFN (5, 10, or 20 *μ*M) or vehicle control (0.05% DMSO) for 40 min, followed by stimulation with NG (5 mM) or HG (25 mM) for additional 90 min. Platelet Δψm dissipation **(A,B)** and PS exposure **(C,D)** were determined using flow cytometry. Platelets were lysed and immunoblotted to detect expression levels of cleaved caspase-9 (37 kDa) **(E)**, and cleaved caspase-3 (17 kDa) **(F)**. Data were assessed by a one-way ANOVA followed by Dunnett’s *t*-test (*n* = 3). *p < 0.05, **p < 0.01 and ***p < 0.001 vs. the vehicle control.

As shown in Figure 1, compared with NG treatment, HG-treated human platelets exhibited significant dissipation of Δψm (94.63 ± 0.09% vs. 83.97 ± 1.98%; *p* < 0.001; Figures 1A,B), and increased PS exposure (2.04 ± 0.57% vs. 10.15 ± 0.43%; *p* < 0.001; Figures 1C,D). These HG-induced effects were attenuated by various concentrations of SFN (5, 10, and 20 μM) (*p* < 0.001). Moreover, SFN (20 μM) favorably downregulated HG-mediated cleavage activation of caspase-9 and caspase-3 (*p* < 0.01) (Figures 1E,F). However, compared with NG treatment, HG exposure did not significantly modulate expression of Bax, Bak, or Bcl-xL in human platelets (Supplementary Figures S1A–C), consistent with prior findings (8). Preincubation with SFN likewise failed to alter expression levels of these apoptosis-related proteins in HG-stimulated platelets (Supplementary Figures S1A–C).

**Figure 2:**
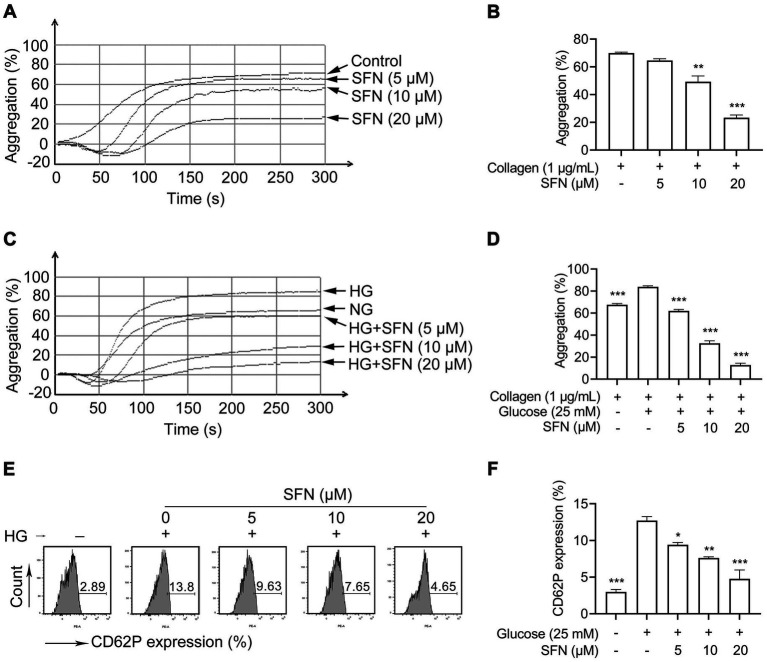
Sulforaphane attenuates HG-induced platelet hyperreactivity. **(A,B)** Washed human platelets were pre-incubated with SFN (5, 10, or 20 μM) or vehicle control (0.05% DMSO) for 40 min. Platelet aggregation was stimulated by 1 μg/mL collagen. Representative aggregation curves **(A)** and aggregation results expressed as maximal amplitude of aggregation **(B)** were shown. **(C–F)** Washed human platelets were pre-incubated with SFN (5, 10, or 20 μM) or vehicle control (0.05% DMSO) for 40 min, followed by stimulation with NG (5 mM) or HG (25 mM) for additional 90 min. **(C,D)** Platelet aggregation was stimulated by 1 μg/mL collagen. **(E,F)** Platelet surface expression of CD62P was analyzed by flow cytometry. Data were assessed by a one-way ANOVA followed by Dunnett’s *t*-test (*n* = 3). *p < 0.05, **p < 0.01 and ***p < 0.001 vs. the vehicle control.

Platelet mitochondrial dysfunction critically contributes to hyperreactivity and thrombotic risk (7). Under normoglycemic conditions, we found that SFN at concentrations of 10 μM and 20 μM showed significant inhibitory effect on collagen-induced platelet aggregation from 69.94 ± 0.72% to 49.27 ± 4.14% (*p* < 0.01) and 23.38 ± 1.92% (*p* < 0.001), respectively (Figures 2A,B). Pre-exposure to HG specifically potentiates collagen-triggered aggregation, but does not affect responses to thrombin or adenosine diphosphate (ADP), indicating collagen-selective hypersensitivity in human platelets (10). Therefore, to examine the ability of SFN to mitigate HG-induced platelet hyperreactivity, we measured platelet aggregation in collagen-activated human platelets. As shown in Figures 2C,D, HG significantly enhanced collagen (1 μg/mL)-induced platelet aggregation compared to NG controls (83.83 ± 0.93% vs. 67.52 ± 1.22%; *p* < 0.001). This potentiation was greatly attenuated by SFN (5–20 μM), reducing aggregation to 61.98 ± 1.28%, 32.55 ± 2.30%, and 12.78 ± 1.64%, respectively (*p* < 0.001). These data confirm SFN not only suppresses basal collagen-induced aggregation (21, 23), but also counteracts HG-potentiated hyperreactivity. Furthermore, HG significantly elevated surface expression of activation marker CD62P from 2.98 ± 0.33% to 12.7 ± 0.55% (*p* < 0.001). SFN treatment (5–20 μM) favorably reduced this expression to 9.41 ± 0.31% (*p* < 0.05), 7.61 ± 0.17% (*p* < 0.01), and 4.76 ± 1.21% (*p* < 0.001) (Figures 2E,F). Collectively, our results demonstrate SFN attenuates HG-induced mitochondrial dysfunction, apoptosis, and hyperreactivity in human platelets *in vitro*.

### 3.2 SFN attenuates HG-induced platelet dysfunction through downregulating AR activity

**Figure 3:**
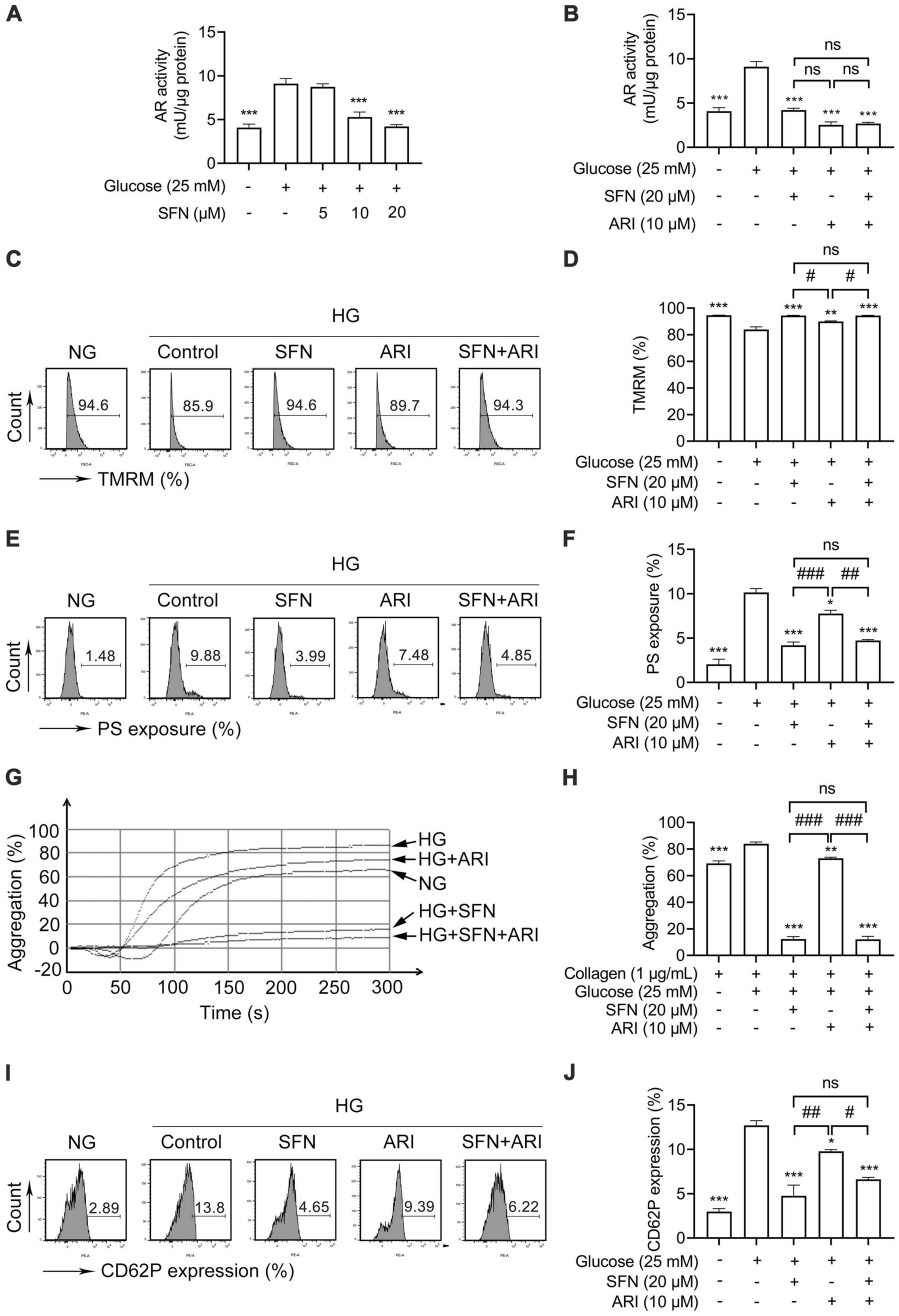
Sulforaphane attenuates HG-induced platelet dysfunction through downregulating AR activity. **(A)** Washed human platelets were pre-incubated with SFN (5, 10, or 20 μM) or vehicle control (0.05% DMSO) for 40 min, followed by stimulation with NG (5 mM) or HG (25 mM) for additional 90 min. AR activity was measured. **(B–J)** Washed human platelets were pre-incubated with SFN (20 μM) with or without a selective AR inhibitor epalrestat (ARI, 10 μM) for 40 min, followed by stimulation with NG or HG for additional 90 min. AR activity was measured **(B)**. Platelet Δψm dissipation **(C,D)** and PS exposure **(E,F)** were determined using flow cytometry. **(G,H)** Platelet aggregation was stimulated by 1 μg/mL collagen. **(I,J)** Platelet surface expression of CD62P was analyzed by flow cytometry. Data were assessed by a one-way ANOVA followed by Dunnett’s *t*-test in **(A)** and by Tukey’s multiple comparisons test in **(B–J)** (*n* = 3). *p < 0.05, **p < 0.01 and ***p < 0.001 vs. the vehicle control; ^#^*p* < 0.05, ^##^*p* < 0.01 and ^###^*p* < 0.001; ns, not significant difference.

The AR catalyzes glucose-to-sorbitol conversion and critically contributes to platelet mitochondrial dysfunction, apoptosis, and hyperreactivity in diabetes (8, 10). We next examined the effect of SFN on AR activity in human platelets in response to HG. As shown in Figure 3A, HG significantly increased AR activity (9.10 ± 0.60 mU/μg protein vs. 4.09 ± 0.39 mU/μg protein; *p* < 0.001), an effect greatly suppressed by SFN (10 and 20 μM) to 5.29 ± 0.57 mU/μg protein and 4.20 ± 0.22 mU/μg protein, respectively (*p* < 0.001). Consistent with this, epalrestat (10 μM), a selective AR inhibitor (ARI), significantly inhibited HG-elevated AR activity (2.54 ± 0.32 mU/μg protein vs. 9.10 ± 0.60 mU/μg protein; *p* < 0.001; Figure 3B). Notably, combining ARI (10 μM) with SFN (20 μM) showed no additive or synergistic inhibition of AR activity (Figure 3B). We further examined whether SFN could bind with human AR by using molecular docking analysis. As shown in Supplementary Figure S2, the docking result showed that the docking score and confidence score of SFN complexed with AR was −119.21 and 0.35, respectively. This indicates that SFN may have a low-affinity binding to AR in human platelets.

We further investigated the role of AR in SFN-mediated attenuation of HG-induced platelet dysfunction. Pretreatment with ARI (10 μM) abrogated HG-induced Δψm dissipation (Figures 3C,D; *p* < 0.01) and PS exposure (Figures 3E,F; *p* < 0.05). Notably, SFN (20 μM) did not further reduce these effects. Similarly, the increases in platelet aggregation (Figures 3G,H) and surface CD62P expression (Figures 3I,J) mediated by HG were also greatly restored by ARI (10 μM) pretreatment. Combining ARI (10 μM) with SFN (20 μM) produced no additive inhibitory effects (Figures 3G–J). These findings indicate that SFN alleviates HG-induced platelet dysfunction primarily through AR downregulation in human platelets *in vitro*.

### 3.3 SFN downregulates AR-mediated ROS/p53 signaling in HG-stimulated human platelets

**Figure 4:**
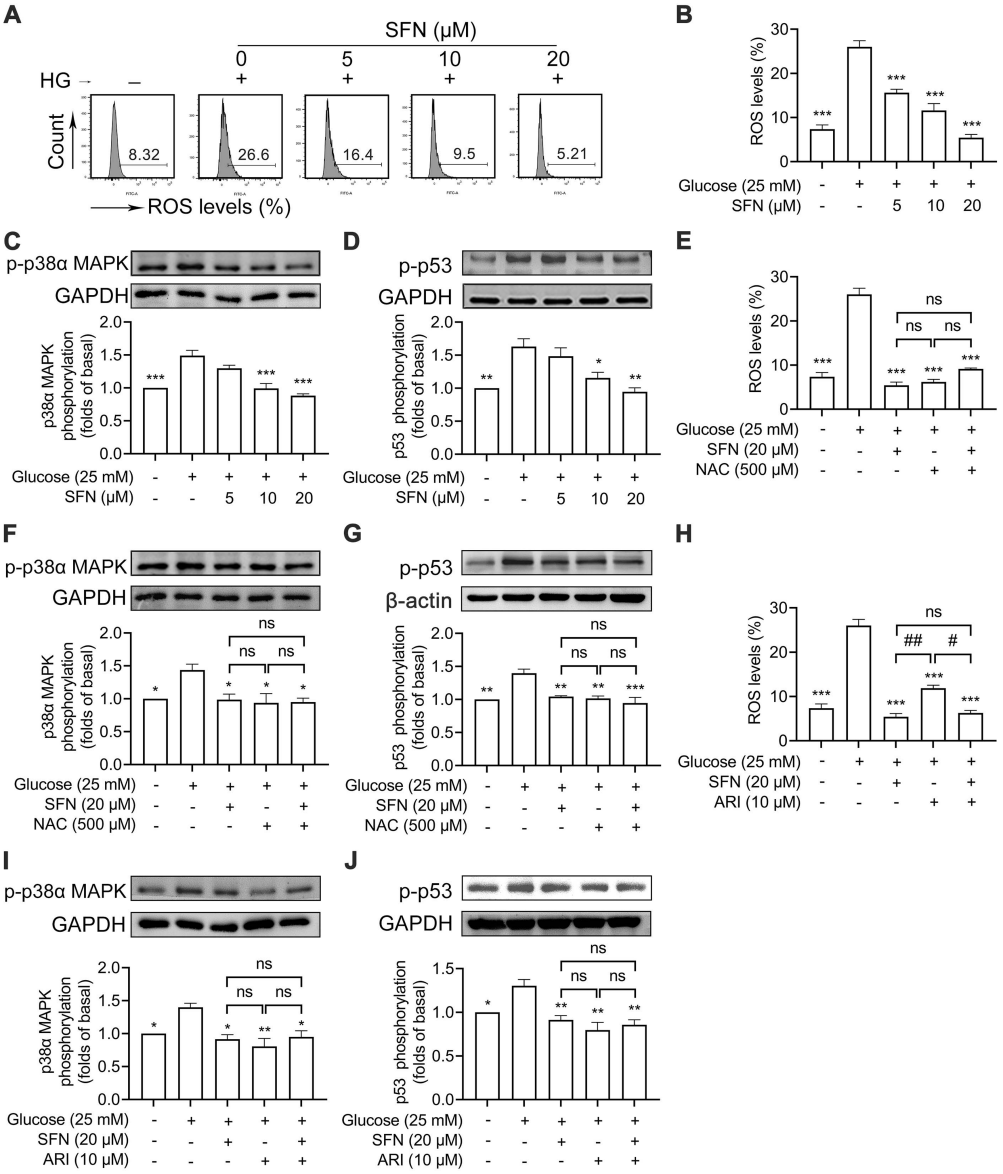
Sulforaphane downregulates AR-mediated ROS/p53 signaling in HG-stimulated human platelets. **(A–D)** Washed human platelets were pre-incubated with SFN (5, 10, or 20 μM) or vehicle control for 40 min, followed by stimulation with NG or HG for additional 90 min. **(A,B)** Intraplatelet ROS levels were measured by flow cytometry. **(C,D)** Platelets were lysed and phosphorylation of p38α MAPK **(C)** and p53 **(D)** were determined by Western blotting. **(E–J)** Washed human platelets were pre-incubated with SFN (20 μM) with or without a ROS scavenger NAC (500 μM) **(E–G)** or ARI (10 μM) **(H–J)** for 40 min, followed by stimulation with NG or HG for additional 90 min. Intraplatelet ROS levels **(E,H)**, and phosphorylation of p38α MAPK **(F,I)** and p53 **(G,J)** were determined. Data were assessed by a one-way ANOVA followed by Dunnett’s *t*-test in **(A–D)** and by Tukey’s multiple comparisons test in **(E–J)** (*n* = 3). *p < 0.05, **p < 0.01 and ***p < 0.001 vs. the vehicle control; ^#^*p* < 0.05 and ^##^*p* < 0.01; ns, not significant difference.

Enhanced ROS generation activates p38α MAPK, leading to increased p53 phosphorylation that promotes platelet dysfunction and thrombus formation in hyperglycemia (8, 10, 26). As shown in Figures 4A,B, HG-induced intraplatelet ROS accumulation was dose-dependently attenuated by SFN (*p* < 0.001). SFN (10 and 20 μM) significantly suppressed HG-stimulated phosphorylation of p38α MAPK (*p* < 0.001) and p53 (*p* < 0.05) (Figures 4C,D). Additionally, the ROS scavenger NAC (500 μM) greatly reversed HG-elevated generation of ROS, and phosphorylation of p38α MAPK and p53. NAC (500 μM) exerted comparable inhibition when compared with those pretreated by SFN (20 μM) (Figures 4E–G). The combination of SFN (20 μM) with NAC (500 μM) did not result in additive inhibitory effects (Figures 4E–G). Having found that decreased AR activity was involved in SFN-attenuated platelet dysfunction in response to HG, we further demonstrated whether the down-regulated ROS/p53 pathway mediated by SFN was dependent on AR activity. As shown in Figures 4H–J, the increases in generation of intraplatelet ROS, and phosphorylation of p38α MAPK and p53 mediated by HG were favorably restored by pretreatment of ARI (10 μM), which did not exhibit additive inhibitory effects when combined with SFN (20 μM). These data demonstrate SFN attenuates the ROS/p53 pathway in HG-stimulated platelets primarily through AR downregulation.

### 3.4 SFN attenuates ROS/p53 signaling through downregulating AR-mediated activation of Src family kinases in HG-stimulated human platelets

**Figure 5:**
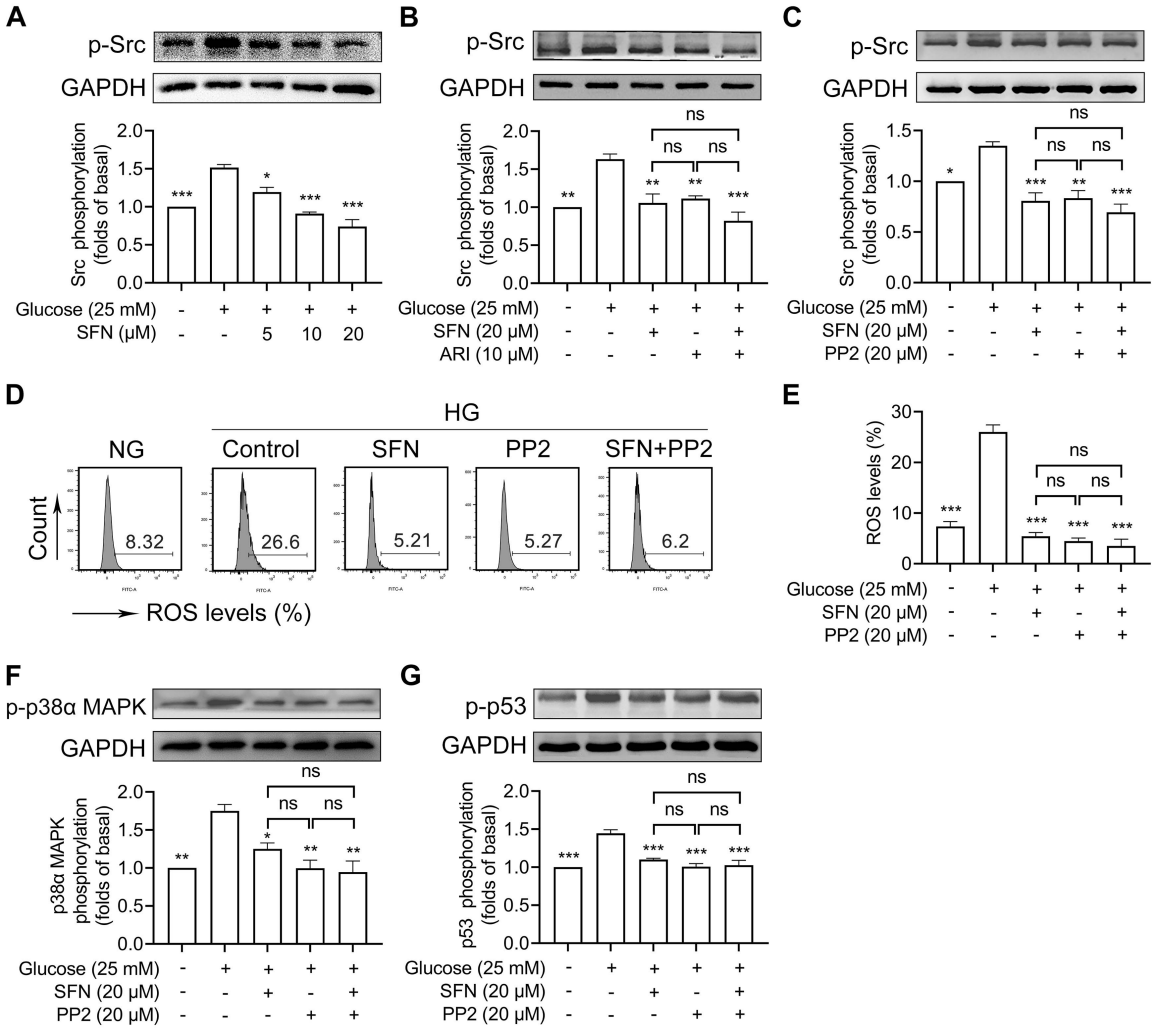
Sulforaphane attenuates ROS/p53 signaling through downregulating AR-mediated activation of Src family kinases in HG-stimulated human platelets. **(A)** Washed human platelets were pre-incubated with SFN (5, 10, or 20 μM) or vehicle control for 40 min, followed by stimulation with NG or HG for additional 90 min. Platelets were lysed and Src phosphorylation was determined by Western blotting. **(B)** Washed human platelets were pre-incubated with SFN (20 μM) with or without ARI (10 μM) for 40 min, followed by stimulation with NG or HG for additional 90 min. Src phosphorylation was determined. **(C–G)** Washed human platelets were pre-incubated with SFN (20 μM) with or without a Src inhibitor PP2 (20 μM) for 40 min, followed by stimulation with NG or HG for additional 90 min. Src phosphorylation **(C)**, intraplatelet ROS levels **(D,E)**, and phosphorylation of p38α MAPK **(F)** and p53 **(G)** were determined. Data were assessed by a one-way ANOVA followed by Dunnett’s *t*-test in **(A)** and by Tukey’s multiple comparisons test in **(B–G)** (*n* = 3). *p < 0.05, **p < 0.01 and ***p < 0.001 vs. the vehicle control; ns, not significant difference.

We previously demonstrated that SFN alleviated collagen/ox-LDL-induced mitochondrial dysfunction, ROS generation, and platelet hyperreactivity primarily through Src kinase downregulation (20, 23). Thus, we intended to determine whether Src kinases mediate the inhibitory effect of SFN on ROS/p53 signaling in HG-stimulated platelets. We found that HG-increased Src phosphorylation was significantly down-regulated by SFN (5–20 μM) treatment (Figure 5A). ARI (10 μM) greatly restored HG-increased Src phosphorylation (*p* < 0.01) and showed no additive inhibitory effects when combined with SFN (20 μM) (Figure 5B). This indicates that Src family kinases are located downstream of AR during SFN treatment. We next validated the role of AR/Src signaling in SFN-mediated attenuation of the ROS/p53 pathway. Pharmacological Src inhibition with PP2 (20 μM) blocked HG-induced Src phosphorylation (*p* < 0.01; Figure 5C), intraplatelet ROS generation (*p* < 0.001; Figures 5D,E), and abrogated phosphorylation of p38α MAPK (*p* < 0.01; Figure 5F) and p53 (*p* < 0.001; Figure 5G). Critically, combining SFN (20 μM) with PP2 (20 μM) produced no synergistic inhibition of these markers (Figures 5C–G). These data collectively demonstrate that SFN suppresses ROS/p53 signaling in HG-stimulated platelets primarily through AR-dependent downregulation of Src kinase activity.

### 3.5 SFN attenuates AR-mediated platelet dysfunction mainly through downregulating Src/ROS/p53 signaling in HG-stimulated human platelets

**Figure 6:**
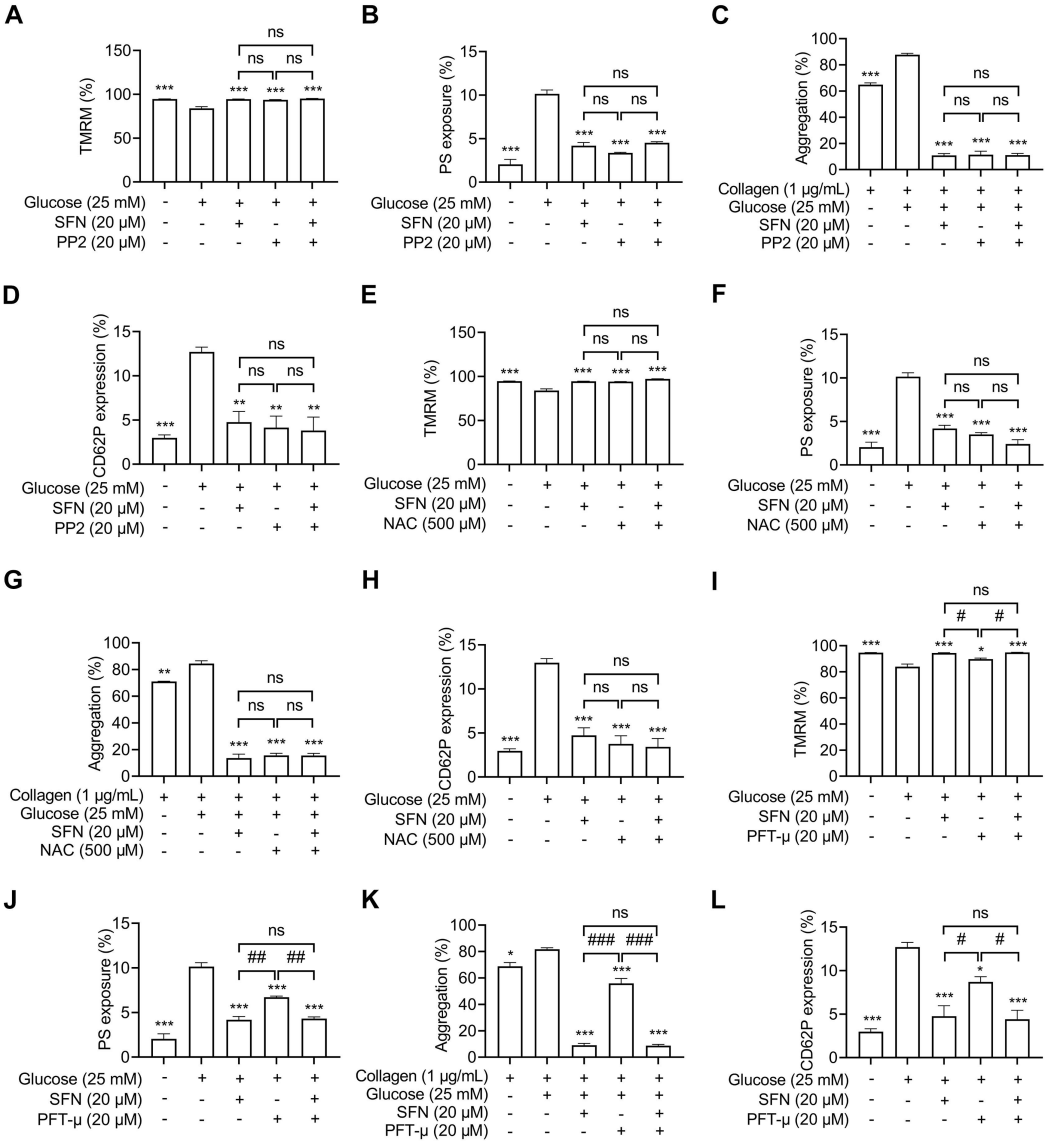
Sulforaphane attenuates AR-mediated platelet dysfunction mainly through downregulating Src/ROS/p53 signaling in HG-stimulated human platelets. Washed human platelets were pre-incubated with SFN (20 μM) with or without PP2 (20 μM) **(A–D)**, NAC (500 μM) **(E–H)**, or a p53 inhibitor PFT-μ (20 μM) **(I–L)** for 40 min, followed by stimulation with NG or HG for additional 90 min. Platelet Δψm dissipation **(A,E,I)** and PS exposure **(B,F,J)** were determined using flow cytometry. Platelet aggregation was stimulated by 1 μg/mL collagen **(C,G,K)**. Platelet surface expression of CD62P was analyzed by flow cytometry **(D,H,L)**. Data were assessed by a one-way ANOVA followed by Tukey’s multiple comparisons test (*n* = 3). *p < 0.05, **p < 0.01 and ***p < 0.001 vs. the vehicle control; ^#^*p* < 0.05, ^##^*p* < 0.01, and ^###^*p* < 0.001; ns, not significant difference.

Having demonstrated SFN-mediated suppression of the Src/ROS/p53 axis in HG-stimulated platelets, we investigated its functional relevance to SFN’s protective effects. Pharmacological inhibition with PP2 (20 μM), NAC (500 μM) or a p53 inhibitor PFT-μ (20 μM) prevented HG-induced Δψm dissipation (Figures 6A,E,I), PS exposure (Figures 6B,F,J), platelet aggregation (Figures 6C,G,K) and surface CD62 expression (Figures 6D,H,L). Critically, combining SFN (20 μM) with any inhibitor showed no additive effects. These data establish that SFN attenuates AR-driven platelet dysfunction primarily via downregulation of the Src/ROS/p53 pathway *in vitro*.

## 4 Discussion

The incidence and mortality rates of CVDs continue to rise annually, posing a major threat to global health (27). T2DM is a significant risk factor for CVDs. Hyperglycemia-induced platelet dysfunction in diabetes constitutes an important pathophysiological basis for associated cardiovascular and cerebrovascular complications, particularly thrombosis (3). Dietary intervention is considered a key approach for preventing and managing T2DM and its association with CVDs (13). Our current study elucidated that SFN, a dietary isothiocyanate enriched in cruciferous vegetables, alleviated HG-induced platelet dysfunction by attenuating platelet mitochondrial dysfunction, apoptosis, and hyperreactivity. The underlying mechanism is mainly through downregulating AR activity, resulting in decreased Src/ROS/p53 signaling pathway. These findings demonstrate for the first time that SFN may confer significant protection against hyperglycemia-associated atherothrombosis by targeting platelet AR-mediated signaling and thereby attenuating aberrant platelet function.

**Figure 7:**
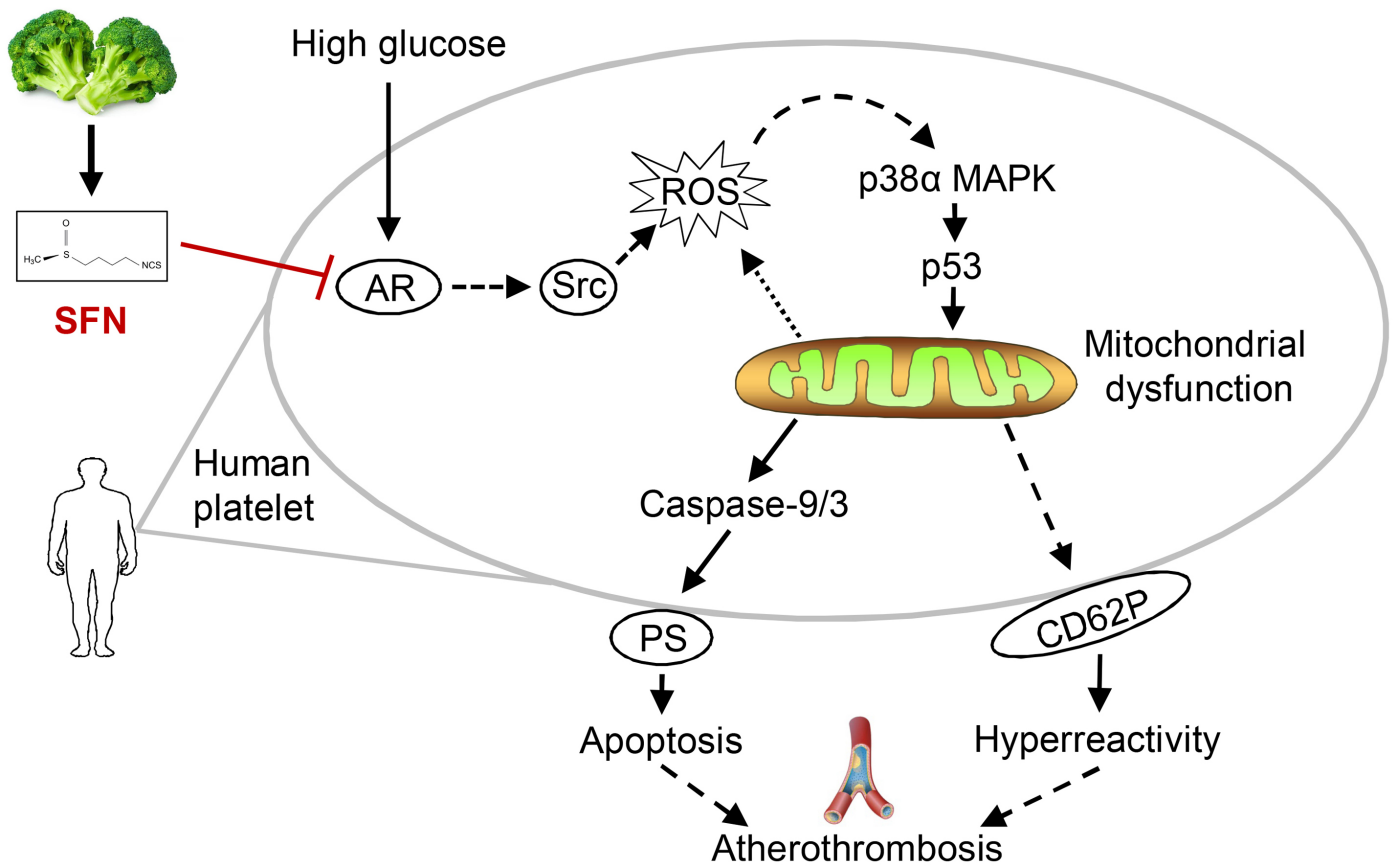
Proposed mechanism of SFN in attenuating platelet dysfunction under HG conditions. SFN alleviates HG-induced platelet mitochondrial dysfunction, apoptosis, and hyperreactivity primarily through suppression of AR activity. This inhibition downregulates the Src/ROS/p53 signaling pathway, ultimately protecting against hyperglycemia-associated atherothrombosis by normalizing platelet function.

Controlling excessive platelet aggregation and activation represents a critical therapeutic strategy for preventing and managing diabetes-associated complications (9). While current antiplatelet agents enable effective thrombolysis and thromboprophylaxis in T2DM, they are associated with significant adverse effects, such as an elevated risk of bleeding complications and drug resistance (9, 28). Abnormal activation of AR and its mediation of signaling pathway in platelets during hyperglycemia contribute to the development of numerous diabetic complications, particularly cardiovascular, cerebrovascular, and thromboembolic events (8, 10). Research on platelet AR activity supports targeting AR signaling as an attractive strategy to prevent platelet hyperactivation (“hypersensitive” phenotype) and inhibit thrombosis, and preserve physiological hemostasis in T2DM (10, 12). We and others have previously shown a protective role of SFN against platelet hyperactivation induced by physiological agonists and hyperlipidemic conditions (20–23). However, whether and how SFN modulates platelet hyperreactivity under hyperglycemic conditions remains unclear. Our current study demonstrated that SFN attenuated AR activity in human platelets exposed to HG. Using the selective AR inhibitor epalrestat, we further showed that SFN improved platelet function primarily by downregulating AR activity and its downstream signaling, including Src family kinases, leading to subsequent inhibition of the ROS/p53 pathway. Thus, through targeting platelet AR and its downstream signaling and attenuating HG-induced platelet dysfunction, SFN may play important protective roles in atherothrombosis under hyperglycemic conditions (Figure 7).

The SFN is widely recognized as a cytoprotective agent targeting intracellular signaling pathways (19). Our computational docking suggested low-affinity binding between SFN and platelet AR (Supplementary Figure S2). Given that platelets are anucleate and lack genomic DNA, their capacity for *de novo* AR protein synthesis *in vitro* remains uncertain and warrants further investigation. Future *in vivo* studies should explore the mechanism underlying SFN-mediated AR inhibition. Key questions include whether this occurs through modulation of *AKR1B1* (the AR-encoding gene) expression or involves glutathionylation-induced changes in AR protein conformation and function. Additionally, elucidating the precise molecular interactions between SFN (or its bioactive metabolites) and the AR ligand-binding domain or co-regulator interfaces represents a critical research priority. Understanding these interactions is imperative for designing next-generation AR modulators with enhanced specificity and reduced off-target effects. Simultaneously, efforts to improve the bioavailability, pharmacokinetic profile, and targeted delivery of SFN or its synthetic analogs are crucial. Strategies such as nanoparticle formulations or prodrug strategies may help bridge the translational gap between promising *in vitro* activity and effective *in vivo* efficacy in relevant preclinical models. Collectively, these focused research avenues, driven by the mechanistic insights reported here, offer significant potential for translating SFN’s AR-modulating properties into clinically impactful therapeutic or nutraceutical interventions.

T2DM is associated with oxidative stress (29). Given that hyperglycemia-induced ROS production and oxidative stress are pathophysiologically linked to T2DM and its complications, therapeutic antioxidants targeting ROS may effectively mitigate disease progression (30). Under hyperglycemic conditions, AR signaling drives robust intraplatelet ROS generation. Enhanced ROS activates p38α MAPK, which increases p53 phosphorylation, inducing mitochondrial dysfunction and damage in diabetic platelets. This cascade critically promotes platelet dysfunction and thrombus formation (8, 10, 26). A ROS scavenger NAC is able to attenuate systemic platelet activation and cerebral vessel thrombosis in diabetes (31). We herein demonstrated that SFN attenuated intraplatelet ROS generation by suppressing AR activity in HG-stimulated human platelets. Using NAC, we further established that SFN inhibited AR-mediated platelet dysfunction under hyperglycemic conditions primarily via suppression of ROS-dependent oxidative stress. These findings align with previous reports from our group and others, indicating that the antioxidant properties of SFN are central to its cardiovascular protective effects (20, 23, 32). However, the *in vivo* effects of SFN supplementation on platelet function in T2DM and its underlying mechanisms warrant further investigation.

The critical role of Src family kinases in platelet activation is well-established (33). Under hyperlipidemic conditions, Src kinase activation induces intraplatelet ROS generation, platelet hyperreactivity, and thrombus formation (34). We previously demonstrated that SFN attenuated collagen-and ox-LDL-induced platelet hyperreactivity by downregulating Src family kinases (20, 35). Glucose synergistically enhances collagen-stimulated platelet activation via increased AR activity (10), indicating that AR activates glycoprotein VI (GPVI)-associated signaling components, including Src kinases and protein kinase C (PKC). Our current study demonstrated that HG significantly induced Src phosphorylation in an AR-dependent manner, consequently attenuating intraplatelet ROS generation and platelet dysfunction in human platelets. Moreover, SFN inhibited collagen-induced platelet aggregation by suppressing AR-and GPVI-mediated Src kinase activation. Using the selective Src inhibitor PP2, we further established that SFN ameliorated HG-induced platelet dysfunction primarily through Src kinase downregulation. These findings indicate that SFN-mediated downregulation of Src kinases may critically contribute to mitigating platelet dysfunction and preventing atherothrombosis under hyperglycemic conditions. In addition, activation of cAMP/PKA pathway mediated by SFN weakens intraplatelet ROS generation, resulting in decreased mitochondrial dysfunction, apoptosis, and platelet hyperreactivity (20, 23). The present study also found that the intracellular cAMP levels were depleted by HG stimulation in human platelets, which were significantly reversed by the treatment of SFN (Supplementary Figure S3). However, the mechanisms by which HG modulates the platelet cAMP/PKA signaling pathway and whether this pathway mediates the ameliorative effects of SFN on platelet dysfunction under hyperglycemia remain poorly understood and warrant further investigation.

Platelet mitochondrial dysfunction and apoptosis represent key pathological features of CVDs (8, 36). These processes critically promote atherothrombosis and accelerate CVD progression (37). Extensive evidence supports a close relationship between platelet activation and apoptosis (38). Both platelet hyperreactivity and apoptosis are observed in platelets from patients with DM (8) and in platelets exposed to ox-LDL or elevated thrombin concentrations (38, 39). These distinct cellular states depend on the severity of mitochondrial damage; mild mitochondrial dysfunction promotes platelet activation, while severe damage drives apoptotic pathways (8). Consistent with prior evidence, our findings demonstrated the coexistence of platelet apoptosis and hyperreactivity in HG-exposed human platelets. HG stimulation induced mild mitochondrial dysfunction. Thus, we suspect that SFN-mediated attenuation of this pathology may concurrently mitigate both apoptosis and hyperreactivity. Collectively, all these effects mediated by SFN appear to synergistically protect against cardiovascular complications. However, given the complex interplay between platelet mitochondrial dysfunction, apoptosis, and hyperreactivity, the multi-target regulatory networks through which SFN operates under hyperglycemic conditions require systematic elucidation.

While this study provides mechanistic insights into SFN’s regulation of AR in platelet dysfunction under high-glucose conditions, several limitations should be acknowledged. First, the exclusive use of *in vitro* platelet models limits direct extrapolation to human pathophysiology, as these systems lack the complex metabolic, hormonal, and vascular interactions present *in vivo*. Additionally, the absence of preclinical validation in diabetic animal models or human clinical samples restricts conclusions regarding clinical applicability. Future studies should prioritize *in vivo* validation and dose–response assessments to bridge these translational gaps.

## 5 Conclusion

Our findings establish a novel direct link between SFN and platelet dysfunction under HG conditions. We demonstrated that SFN effectively ameliorated AR-mediated platelet mitochondrial dysfunction, apoptosis, and hyperreactivity, primarily through inhibition of the Src/ROS/p53 signaling pathway. Therefore, these findings may offer new insights for the development of functional food ingredients derived from natural sources to protect against atherothrombosis in patients at risk of CVDs, such as diabetes mellitus.

## Data Availability

The original contributions presented in the study are included in the article/Supplementary material, further inquiries can be directed to the corresponding author.
