# Supplementary material for: Sulforaphane attenuates aldose reductase-mediated platelet dysfunction in high glucose-stimulated human platelets via downregulation of the Src/ROS/p53 signaling pathway
Source: Front Nutr. 2025 Aug 1;12:1663245. doi: 10.3389/fnut.2025.1663245 (PMC12353706; doi:10.3389/fnut.2025.1663245)
Supplement: Supplementary file 1 [file Table_1.docx]

***Supplementary Material***

**Sulforaphane Attenuates Aldose Reductase-mediated Platelet Dysfunction in High Glucose-stimulated Human platelets via** **Downregulation of the Src/ROS/p53 Signaling Pathway**

Xiaoyan Bi^1†^, Xinhui Huang^2†^, Chunmei Zhang^3†^, Xin Zhao^1^, Junyu Ma^1^, Mengyao Li^1^, Xuexun Li^1^, Bangzhao Zeng^1^, Rong Li^1^, Xian Zhang^4^, Fuli Ya^1*^

^1^Department of Nutrition, School of Public Health, Dali University, Dali, Yunnan Province 671000, China;

^2^Huzhou Health Vocational College, Huzhou, Zhejiang Province 313000, China;

^3^Department of Laboratory Teaching Center, School of Public Health, Dali University, Dali, Yunnan Province 671000, China;

^4^Department of Cardiology, Dali Bai Autonomous Prefecture People's Hospital, Dali, Yunnan Province 671000, China;

^†^Xiaoyan Bi, Xinhui Huang, and Chunmei Zhang contributed equally to this work.

***Corresponding author:**

Fuli Ya, MD, Ph.D

Department of Nutrition, School of Public Health, Dali University,

No. 22, Wanhua Road, Dali, Yunnan Province, PR China 671000;
E-mail: yafuli@yeah.net; Tel/Fax: 86-872-2257382

**1 Supplementary materials and methods**

***1.1 Molecular docking analysis***

Protein-ligand docking was performed using HDOCK (version 3.0) to predict the binding mode of SFN to human AR (PDB ID: 2R24). The crystal structure of AR (resolution: 1.75 Å) was retrieved from the RCSB Protein Data Bank. Prior to docking, the receptor was prepared by removing water molecules and heteroatoms, Adding polar hydrogens and assigning CHARMM36 force field charges, and defining the binding site as a 15-Å grid centered on the catalytic Zn²⁺ ion. The 3D structure of L-sulforaphane was energy-minimized using Gaussian 16 at the B3LYP/6-31G level. The top-ranked pose based on HDOCK score (energy units: kcal/mol) was selected for subsequent analysis. Validation was performed by re-docking the co-crystallized ligand (RMSD < 1.0 Å).

***1.2 Measurement of platelet intracellular cAMP levels in human platelets***

To determine the levels of intracellular cyclic adenosine monophosphate (cAMP), a commercial cAMP Direct Immunoassay Kit (Cloud-Clone Corp., Wuhan, China) was used, according to the manufacturer’s protocols and our previously described method (1, 2). In brief, human washed platelets (2.0×10^8^ platelets/mL) were pre-incubated with different concentrations of SFN (5, 10, or 20 μM) or vehicle control (0.05% DMSO) for 40 min at 37°C, followed by the stimulation of normal glucose (NG; 5 mM) or high glucose (NG; 25 mM) for additional 90 min. Platelets were then lysed with HCl (100 mM) for 20 min on ice and the levels of cAMP in the supernatant were detected.

**2 Supplementary references**

1. Zhou XY, Huang XH, Wu CT, Ma YJ, Li WQ, Hu JQ, et al. Sulforaphane Attenuates Glycoprotein VI-mediated Platelet Mitochondrial Dysfunction through Up-regulating cAMP/PKA Signaling Pathway in vitro and in vivo. *Food Funct*. (2023) 14: 3613-3629. doi: 10.1039/D2FO03958C

2. Li, WQ, Wu CT, Zhou XY, Huang XH, Zhang CM, Ma YJ, et al. Sulforaphane Attenuates CD36-mediated Platelet Hyperreactivity through Modulating cAMP/PKA/NOX2 Signaling in Hyperlipidemic Conditions. *Food Sci Hum Wellness*. (2025) 14: 9250165. doi: 10.26599/FSHW.2024.9250165

**3 Supplementary figures and legends**

**
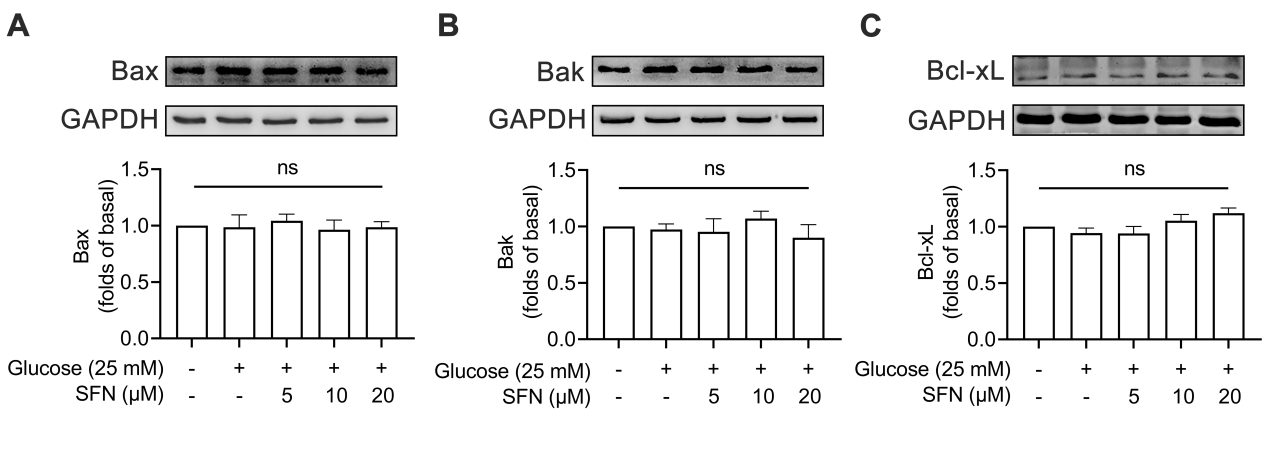
**

**Supplementary Fig. S1. Sulforaphane (SFN) did not significantly modulate expression levels of** **Bax, Bak, and Bcl-xL in human platelets in response to high glucose (HG).** Washed human platelets were pre-incubated with SFN (5, 10, or 20 µM) or vehicle control (0.05% DMSO) for 40 min, followed by stimulation with NG (5 mM) or HG (25 mM) for additional 90 min. Platelets were lysed and expression levels of Bax **(A)**, Bak **(B)**, and Bcl-xL **(C)** were determined by Western blotting. Data were presented as mean ± standard error of the mean (SEM) and assessed by a one-way analysis of variance (ANOVA) followed by Dunnett’s *t*-test. ns, not significant difference.


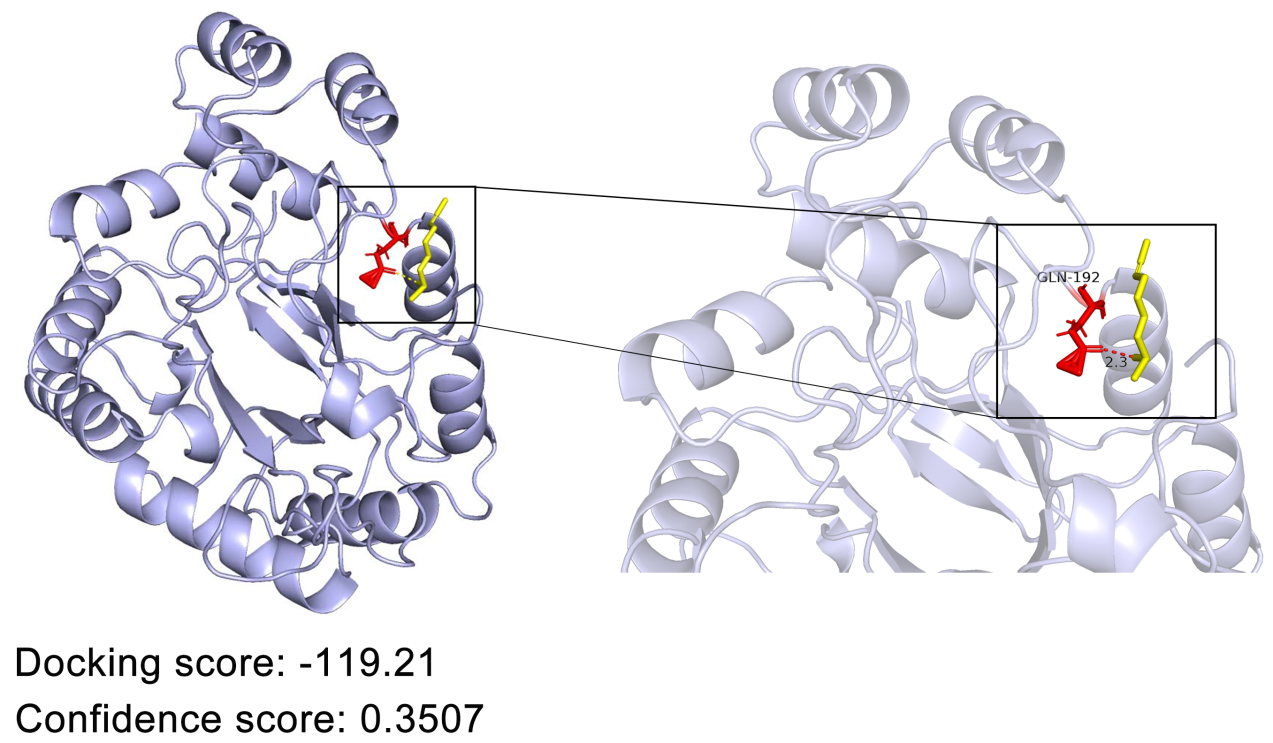


**Supplementary Figure S2. Molecular docking analysis of the interaction between SFN and human aldose reductase.**

**
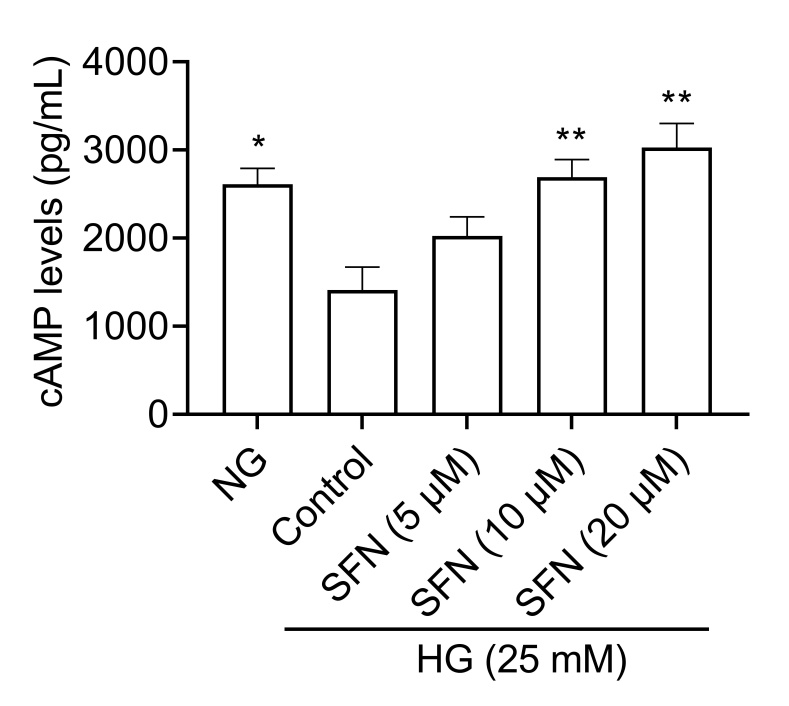
**

**Supplementary Figure S3. SFN significantly increased intraplatelet cAMP levels in human platelets in response to HG.** Washed human platelets were pre-incubated with SFN (5, 10, or 20 µM) or vehicle control (0.05% DMSO) for 40 min, followed by stimulation with NG (5 mM) or HG (25 mM) for additional 90 min. Platelets were lysed and cAMP levels were determined. Data were presented as mean ± SEM and assessed by a one-way ANOVA followed by Dunnett’s *t*-test. **P* < 0.05 and ***P* < 0.01 *vs.* the vehicle control.
